# RNA sequencing of kidney and liver transcriptome obtained from wild cynomolgus macaque *(Macaca fascicularis*) originating from Peninsular Malaysia

**DOI:** 10.1186/s13104-018-4014-1

**Published:** 2018-12-22

**Authors:** Joey Ee Uli, Christina Seok-Yien Yong, Swee Keong Yeap, Noorjahan Banu Alitheen, Jeffrine J. Rovie-Ryan, Nurulfiza Mat Isa, Soon Guan Tan

**Affiliations:** 1 Department of Cell and Molecular Biology, Faculty of Biotechnology and Biomolecular Sciences, Universiti Putra Malaysia, Serdang, Selangor Malaysia; 2 Department of Biology, Faculty of Science, Universiti Putra Malaysia, 43400 Serdang, Selangor Malaysia; 3 China-ASEAN College of Marine Sciences, Xiamen University, Sepang, Selangor Malaysia; 4 National Wildlife Forensic Laboratory, Ex-Situ Conservation Division, Department of Wildlife and National Parks, Kuala Lumpur, Malaysia

**Keywords:** *Macaca fascicularis*, Cynomolgus macaque, RNA sequencing, Transcriptome, Biomedical science, Kidney, Liver

## Abstract

**Objective:**

Using high-throughput RNA sequencing technology, this study aimed to sequence the transcriptome of kidney and liver tissues harvested from Peninsular Malaysia cynomolgus macaque (*Macaca fascicularis*). *M. fascicularis* are significant nonhuman primate models in the biomedical field, owing to the macaque’s biological similarities with humans. The additional transcriptomic dataset will supplement the previously described Peninsular Malaysia *M. fascicularis* transcriptomes obtained in a past endeavour.

**Results:**

A total of 75,350,240 sequence reads were obtained via Hi-seq 2500 sequencing technology. A total of 5473 significant differentially expressed genes were called. Gene ontology functional categorisation showed that cellular process, catalytic activity, and cell part categories had the highest number of expressed genes, while the metabolic pathways category possessed the highest number of expressed genes in the KEGG pathway analysis. The additional sequence dataset will further enrich existing *M. fascicularis* transcriptome assemblies, and provide a dataset for further downstream studies.

**Electronic supplementary material:**

The online version of this article (10.1186/s13104-018-4014-1) contains supplementary material, which is available to authorized users.

## Introduction

Cynomolgus macaques (*Macaca fascicularis*) are nonhuman primate (NHP) models significant to biomedicine due to their close evolutionary relationship with humans. The cynomolgus macaque’s recapitulation of human physiology, genetics, and behaviour is advantageous as translational models for various studies in the biomedical field, including drug development and safety testing [1, 2]. Cynomolgus macaque individuals from different geographical locations have been shown to exhibit genetic characteristics that varies between geographical origins [3]. Therefore, it is vital that the genomes and transcriptomes of cynomolgus macaque NHP models originating from different geographical locations are sequenced as a reference for future biomedical research design and implementation.

This study describes the RNA sequencing (RNA-seq) of two tissues—kidney and liver—harvested from wild Peninsular Malaysian cynomolgus macaques, and is an extension of a previous study [4] whereby the lymph node, spleen, and thymus transcriptomes of wild Peninsular Malaysian *M. fascicularis* were also sequenced with RNA-seq technology. An additional 75,350,240 sequence reads were obtained from this study, supplementing the previous wild Peninsular Malaysian *M. fascicularis* dataset for downstream applications. Furthermore, additional Malaysian cynomolgus macaque RNA-seq datasets will further furnish the cynomolgus macaque genome and transcriptome annotations and also provide a valuable asset for biomedical studies involving cynomolgus macaques.

## Main text

### Materials and methods

#### Samples

Two male conflict macaques that appeared to belong in the same family group were captured by the Department of Wildlife and National Parks (DWNP) from the state of Selangor. Visual examination showed that the macaques were free of disease. Euthanisation and harvesting of organs were also performed by authorised and qualified veterinarians of DWNP. In brief, the macaques were sedated intramuscularly using general anaesthesia (combination of ketamine, 5–10 mg/kg and xylazine, 0.2–0.4 mg/kg) before a lethal dosage of Dolethal^®^ were given intravenously. Harvested kidney and liver tissues were stored in RNAlater RNA Stabilization Agent (Qiagen). Total RNA extraction was carried out with RNeasy Mini Kit (Qiagen). A modified protocol was employed to remove genomic DNA contamination by incorporating Epicentre’s DNase I solution. RNA extract integrity was determined using 2100 Bioanalyzer (Agilent Technologies, Inc., United States of America) via RNA Pico chip. Samples with RIN > 7.0 were selected for library construction.

#### High-throughput sequencing, data analysis, and validation

Preparation of *M. fascicularis* kidney and liver RNA-seq libraries followed methods previously described in Ee Uli et al. [4]. Raw sequencing reads were submitted to NCBI Short Read Archive under accessions SRX2499144-SRX2499147. For data analyses, we used the same bioinformatic workflow and tools as depicted in our earlier publication [4]. Briefly, the assembly of raw sequence reads, normalisation, empirical analysis of differentially expressed genes (EDGE test), and gene ontology (GO) and KEGG pathway annotations were performed in CLC Genomics Workbench (CLCGW) version 7.5.1 (Qiagen Denmark), while functional classification of expressed genes in the kidney and liver transcriptomes was performed using Panther Database version 11.1 released on 2016-10-24 (http://pantherdb.org/) and DAVID Bioinformatics Resources version 6.8 (https://david-d.ncifcrf.gov/home.jsp). To better present the tissue-specific genes, we combined gene expression data from this study and our previous study [4] to create a Venn diagram representation of tissue-specific genes using the web tool Venn Diagrams (http://bioinformatics.psb.ugent.be/webtools/Venn). Validation of the RNA-seq dataset was performed with NanoString nCounter Elements XT (NanoString Technologies Inc., Seattle, WA, USA) gene expression assay. The methodology was the same as previously described in Ee Uli et al. [4]. Additional file 1 lists the genes utilised for the RNA-seq validation and their respective probe pair sequences.

### Results

#### Sequence reads trimming and RNA-seq mapping

The number of raw kidney and liver sequence reads obtained were 44,452,083 and 30,898,157 respectively, each read possessing a sequence length of 70 bp. After trimming, the number of kidney and liver sequence reads were 41,854,334 and 28,943,585 singly. Summing up to a total of 70,788,919 good quality reads retained for assembly, with the average lengths of the reads ranging from 58.8 to 60.1 bp. Post-trimming, 93.94% of the reads were retained and were considered suitable for RNA-seq mapping. The overall mapping percentage of the sequence reads for each RNA-seq library ranged from 65.00 to 68.59%. Additional file 2 summarises the statistics of the sequence reads trimming and mapping.

#### Normalisation and differential gene expression analysis

A total of 5473 significant differentially expressed genes were called. Additional file 3 lists the differentially expressed genes identified from the EDGE test with their respective annotated gene names and descriptions. Within the Kidney vs. Liver tissue comparison, the top three most upregulated genes in liver were *PLA2G2A*, *SAA1*, and *ORM1*, while the three most downregulated genes include *UMOD*, *TINAG*, and *DHDH*.

#### Tissue-specific genes

**Fig. 1:**
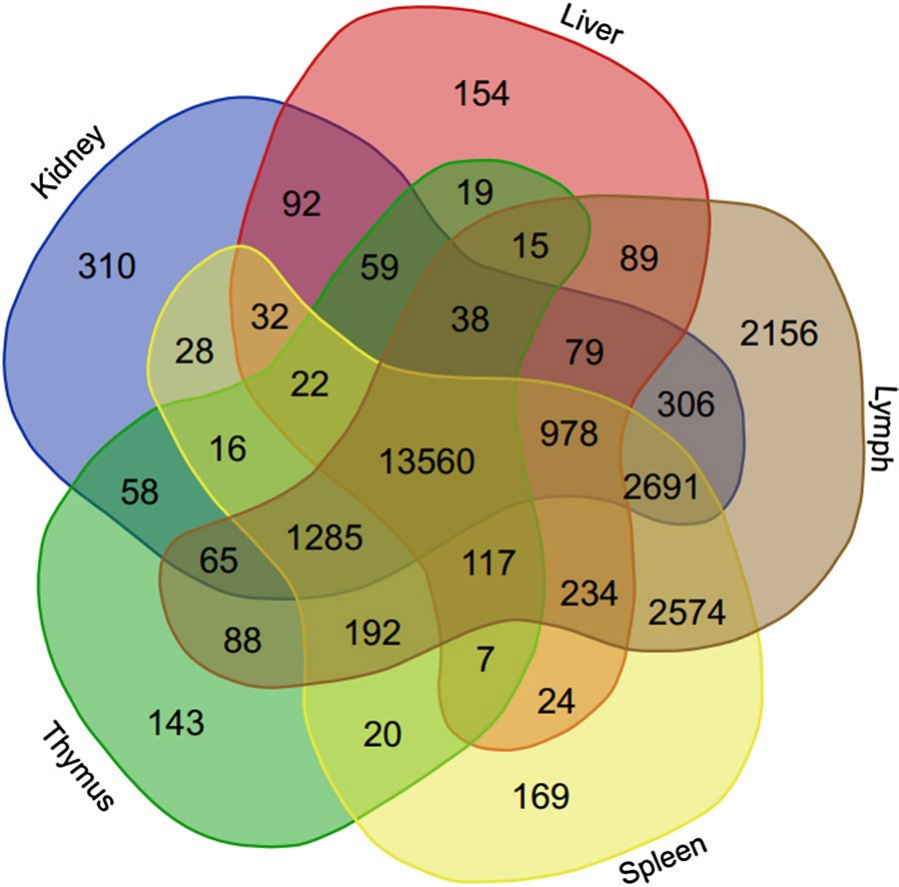
Venn diagram of the number of expressed genes (normalised expression value > 1) in their respective tissues

Using gene expression data obtained from our previous study [4] and this study, a Venn diagram representation of tissue-specific genes in kidney, liver, lymph node, spleen, and thymus tissues was generated (Fig. 1). Lymph node showed the highest number of tissue-specific genes (2156 genes) among the five tissues compared; while kidney and liver had 310 and 154 tissue-specific genes respectively. The three most highly expressed tissue-specific genes in the kidney include *MIOX*, *AQP2*, and *CDH16*, while the top three tissue-specific genes expressed in the liver include *APCS*, *APOA2*, and LOC102121341. A complete list of tissue-specific genes can be found in Additional file 4.

#### Functional annotation and classification

In the kidney, 14,001, 7046, and 5635 expressed genes were categorised to biological process, molecular function, and cellular component domains respectively, while 12,004, 6059, and 4935 expressed genes in the liver were categorised to BP, MF, and CC domains respectively. The distribution of the expressed genes into different level 2 GO categories are shown in Table 1. The BP category with the highest number of expressed genes for both tissues is cellular process (kidney, 4039; liver, 3496). For the MF domain, the catalytic activity category contains the highest number of expressed genes for both tissues (kidney, 2709; liver, 2419). As for CC, both tissues show the highest number of expressed genes in the cell part category (kidney, 2185; liver, 1954).

**Table 1:** Categorisation of expressed genes to gene ontology categories in kidney and liver tissues

Gene ontology	Number of genes	
	Kidney	Liver
Biological process		
Cellular process	4039	3496
Metabolic process	3493	3114
Localization	1060	926
Response to stimulus	1031	878
Developmental process	939	745
Cellular component organization or biogenesis	842	753
Biological regulation	923	740
Multicellular organismal process	668	499
Immune system process	499	443
Biological adhesion	247	211
Reproduction	165	120
Locomotion	81	71
Rhythmic process	6	1
Growth	4	4
Cell killing	4	3
Molecular function		
Catalytic activity	2709	2419
Binding	2660	2310
Transporter activity	572	435
Receptor activity	446	348
Structural molecule activity	450	384
Signal transducer activity	147	110
Translation regulator activity	32	31
Antioxidant activity	14	11
Channel regulator activity	16	11
Cellular component		
Cell part	2185	1954
Organelle	1359	1220
Membrane	851	676
Macromolecular complex	744	681
Extracellular region	318	264
Extracellular matrix	96	75
Cell junction	50	39
Synapse	32	26

In the kidney and liver, a total of 4406 and 3960 genes were assigned to KEGG pathway categories respectively. The metabolic pathways category contains the highest number of expressed genes for both kidney and liver tissues—903 and 839 genes respectively. The distribution of the expressed genes into different KEGG pathway categories are shown in Table 2.

**Table 2:** Categorisation of expressed genes to KEGG pathway categories in kidney and liver tissues

KEGG pathway	Number of genes	
	Kidney	Liver
Metabolism		
Global overview and maps	903	1046
mcf01100: metabolic pathways	903	839
mcf01230: biosynthesis of amino acids	0	58
Carbohydrate metabolism	55	37
mcf00520: amino sugar and nucleotide sugar metabolism	0	37
mcf00562: inositol phosphate metabolism	55	0
Energy metabolism	139	135
mcf00190: oxidative phosphorylation	139	135
Glycan biosynthesis and metabolism	0	35
mcf00510: *N*-glycan biosynthesis	0	35
Genetic information processing		
Folding, sorting and degradation	124	119
mcf04141: protein processing in endoplasmic reticulum	124	119
Environmental information processing		
Membrane transport	37	0
mcf02010: ABC transporters	37	0
Signal transduction	600	128
mcf04010: MAPK signalling pathway	180	0
mcf04012: ErbB signalling pathway	69	67
mcf04015: Rap1 signalling pathway	154	0
mcf04022: cGMP-PKG signalling pathway	119	0
mcf04064: NF-kappa B signalling pathway	0	61
mcf04668: TNF signalling pathway	78	0
Signalling molecules and interaction	177	0
mcf04512: ECM-receptor interaction	71	0
mcf04514: cell adhesion molecules (CAMs)	106	0
Organismal system		
Development	101	0
mcf04360: axon guidance	101	0
Immune system	154	199
mcf04610: complement and coagulation cascades	0	56
mcf04611: platelet activation	101	89
mcf04662: B cell receptor signalling pathway	53	54
Nervous system	172	88
mcf04722: neurotrophin signalling pathway	93	88
mcf04725: cholinergic synapse	79	0
Endocrine system	302	221
mcf04910: insulin signalling pathway	106	98
mcf04915: estrogen signalling pathway	80	73
mcf04917: prolactin signalling pathway	0	50
mcf04921: oxytocin signalling pathway	116	0
Cellular processes		
Transport and catabolism	250	325
mcf04142: lysosome	0	86
mcf04144: endocytosis	182	174
mcf04146: peroxisome	68	65
Cellular community—eukaryotes	168	152
mcf04510: focal adhesion	168	152

#### Validation of RNA-seq differential gene expression

Thirteen genes were selected to validate the RNA-seq differential gene expression dataset via NanoString gene expression assay. Normalised fold change values between the RNA-seq and NanoString platforms show concordance in magnitude directionality (Additional file 5). Our previous study also employed the NanoString gene expression assay to validate the RNA-seq dataset, and has shown similar concordance between the two platforms [4].

### Discussion

In our previous endeavour [4], the lymph node, spleen, and thymus transcriptomes of the Malaysian cynomolgus macaque were sequenced via Illumina Hi-seq 2500 technology. The dataset presented in this study provides additional transcriptomic sequences from the kidney and liver of the Malaysian cynomolgus macaque, with a sum of 75,350,240 sequence reads obtained from four tissue libraries. In Ee Uli et al. [4], the transcriptome sequences were derived from immune-related tissues, whereas the kidney and liver transcriptomes generated in this study were obtained from metabolism-related tissues, and to a certain extent, these tissues also pertain to immune functions as well. Cynomolgus macaques are utilised as NHP models for liver-expressed drug metabolising enzyme studies, for example, the pharmacokinetics of the cytochrome P450 family of enzymes have been extensively studied in cynomolgus macaques [2, 5, 6].

It was observed that the percentages of the processed sequence reads that mapped back to the reference genome for all four libraries were below 70%. To rule out sequence contamination, we performed a BLAST alignment of the unmapped reads to NCBI’s nucleotide (nt) database and classified the hits into their specific taxonomic classification. The majority of the unmapped reads were assigned to Cercopithecidae and other primate family taxa, while the remaining reads were unassigned or did not have any BLASTN hits (Additional file 6). This suggests the absence of contaminating sequences, and also suggests the presence of novel transcripts in the kidney and liver dataset of the Malaysian cynomolgus macaque, which are yet to be described and curated in the *M. fascicularis* reference genome.

Comparisons were made between the kidney and liver tissues due to the similarities in metabolic function between the two tissues. However, the specific metabolic functions of both kidney and liver tissues are ultimately different. For instance, the KEGG pathway enrichment analysis highlights 58 liver genes that were specifically enriched in the biosynthesis of amino acids pathway, while 55 kidney genes were involved in the Inositol phosphate metabolism pathway.

Based on the differential gene expression analysis of kidney and liver tissues, the top three most upregulated genes in liver were *PLA2G2A*, *SAA1*, and *ORM1*, while the three most downregulated genes in kidney include *UMOD*, *TINAG*, and *DHDH*. The *PLA2G2A* gene codes for phospholipase A2 group IIA, and is suggested to be involved in the metabolism of phospholipids, inflammatory responses, and antimicrobial defence [7]. *SAA1* codes for the acute-phase serum amyloid A1 protein, and is involved in inflammation response [8]. *ORM1* codes for orosomucoid 1, which is suggested to induce the conversion of monocytes to M2b macrophages in response to type 2 cytokines in cancer patients [9]. *UMOD* codes for uromodulin, a urinary protein which inhibits the crystallisation of renal fluids that causes renal stone formation, and also expedites transepithelial migration of neutrophils in inflammation responses [10]. Tubulointerstitial nephritis antigen is a glycoprotein coded by *TINAG*, which is a nephritogenic antigen implicated in tubular homeostasis and cell survival in the kidney [11]. *DHDH* codes for the enzyme dihydrodiol dehydrogenase, and is suggested to be involved in detoxification processes in the kidney and liver [12].

In the GO analysis, both kidney and liver transcriptomes were observed to have the majority of genes represented by the same categories in their respective GO domains. In the BP domain, the highly represented category was cellular process, while the category with the highest gene representation in the MF domain was catalytic activity. As for CC domain, the highest number of expressed genes was represented in the cell part category. The representation of functional GO categories in the *M. fascicularis* transcriptome were observed to be similar to that of other chordates, including that of the silver carp [13], pig [14], black grouse [15], and pufferfish [16], and suggest a fair representation of the *M. fascicularis* transcriptome with that of other chordates.

The Malaysian cynomolgus macaque, *M. f. fascicularis*, is a unique subspecies that is suggested to have high nucleotide diversity and is genetically unique compared to other South East Asian *M. fascicularis* populations [17–19]. Genetic heterogeneity is suggested to contribute to varied responses in cynomolgus macaques towards drugs and pathogens [20], making research into the Malaysian population of cynomolgus macaque invaluable to the biomedicine field. The transcriptome dataset of the Peninsular Malaysian cynomolgus macaque obtained from this study can be compared with cynomolgus macaques originating from other geographical locations including Mauritius, the Philippines, Indochina, Indonesia, and China. Such comparisons can be utilised for the detection of single nucleotide polymorphisms in the macaque transcriptome, to infer phylogeny utilising a larger set of genic sequences, and also to determine the nucleotide diversity of the Peninsular Malaysian cynomolgus macaque. Furthermore, the transcriptomic data obtained from this study may be utilised to design genic molecular markers for rapid assessment of wild populations of cynomolgus macaques in Peninsular Malaysia in a relatively shorter period of time and at a lower cost.

## Conclusion

Via the Illumina Hi-seq 2500 platform, 75,350,240 sequence reads in total were obtained from kidney and liver transcriptomes of the wild Peninsular Malaysian cynomolgus macaque. Additional sequence reads from the Malaysian cynomolgus macaque individuals will potentially further supplement the present genomic and transcriptomic data of this significant NHP primate model.

## Limitations

- Only two biological replicates were available for library construction and data analyses.
- RNA sequencing of additional tissues will provide more robust transcriptomic dataset of wild Peninsular Malaysia *M. fascicularis*.

## Additional files

**Additional file 1.** Selected genes for NanoString nCounter XT Gene Expression assay validation of RNA-Seq gene expression profiles. Table lists selected genes utilised for the validation of RNA-Seq gene expression profiles via NanoString nCounter XT Gene Expression assay and their respective probes nucleotide sequences.

**Additional file 2.** *Macaca fascicularis* kidney and liver sequence reads trimming and reference guided assembly summary statistics. Summary of *Macaca fascicularis* kidney and liver sequence reads quality trimming and reference guided assembly.

**Additional file 3.** List of differentially expressed genes from kidney vs. liver comparison. Differentially expressed genes in kidney and liver tissues called from the empirical differential gene expression analysis in CLC Genomics Workbench. Listed are the genes with their corresponding fold change values, together with their gene ontology and pathway annotations.

**Additional file 4.** Tissue-specific genes (sorted from highest to lowest Normalised mean expression value). A list of tissue-specific genes generated from a Venn diagram comparison of expressed genes (normalised expression value > 1) in kidney, liver, lymph node, spleen, and thymus tissues.

**Additional file 5.** Validation of RNA-seq differential gene expression results. Log_2_ transformed fold change values were obtained from RNA-seq and NanoString nCounter XT platforms. Black bar represents fold change values obtained from RNA-Seq platform, while white bar represents fold change value obtained from NanoString nCounter XT platform.

**Additional file 6.** BLAST alignment of unmapped *Macaca fascicularis* reads to NCBI nucleotide database. Graphical representation of unmapped *M. fascicularis* sequence reads assigned to taxonomic ranks based on BLAST alignment with NCBI nucleotide (nt) database. Intensity of the colour green represents the number of unmapped reads mapped to a respective taxonomic rank—the more intense the green, the higher the number of reads assigned to a particular taxon.

## Abbreviations

NHP: nonhuman primate
DWNP: Department of Wildlife and National Parks
RNA-seq: RNA sequencing
CLCGW: CLC Genomics Workbench
TPM: transcripts per million
EDGE: empirical analysis of differentially expressed genes
GO: gene ontology
BP: biological process
MF: molecular function
CC: cellular component
