# Supplementary figures and images for: RNA sequencing of kidney and liver transcriptome obtained from wild cynomolgus macaque (Macaca fascicularis) originating from Peninsular Malaysia
Source: BMC Res Notes. 2018 Dec 22;11:923. doi: 10.1186/s13104-018-4014-1 (PMC6303865; doi:10.1186/s13104-018-4014-1)

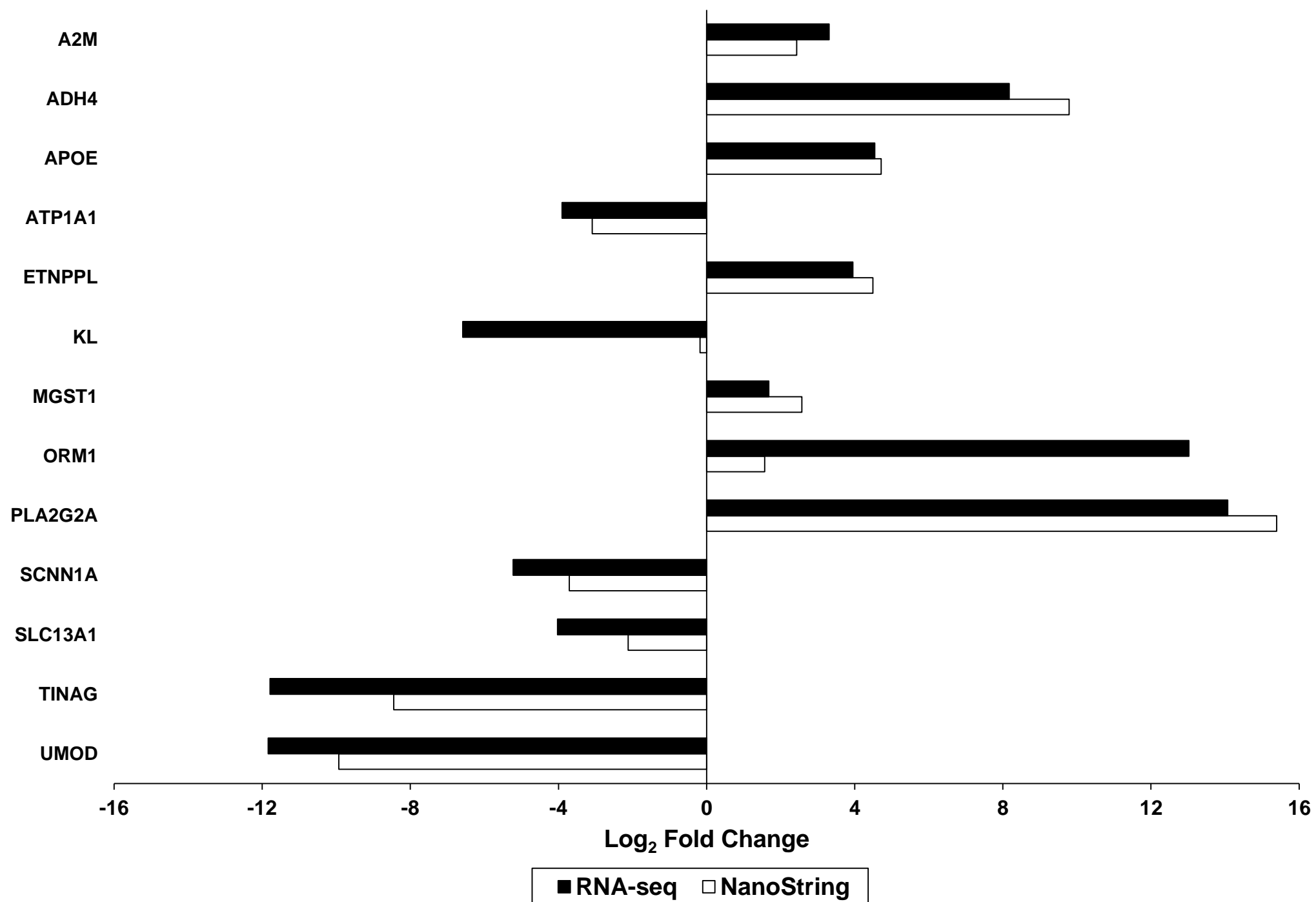

Supplement: Supplementary file 5 — Additional file 5. Validation of RNA-seq differential gene expression results. Log2 transformed fold change values were obtained from RNA-seq and NanoString nCounter XT platforms. Black bar represents fold change values obtained from RNA-Seq platform, while white bar represents fold change value obtained from NanoString nCounter XT platform. [file 13104_2018_4014_MOESM5_ESM.pdf]

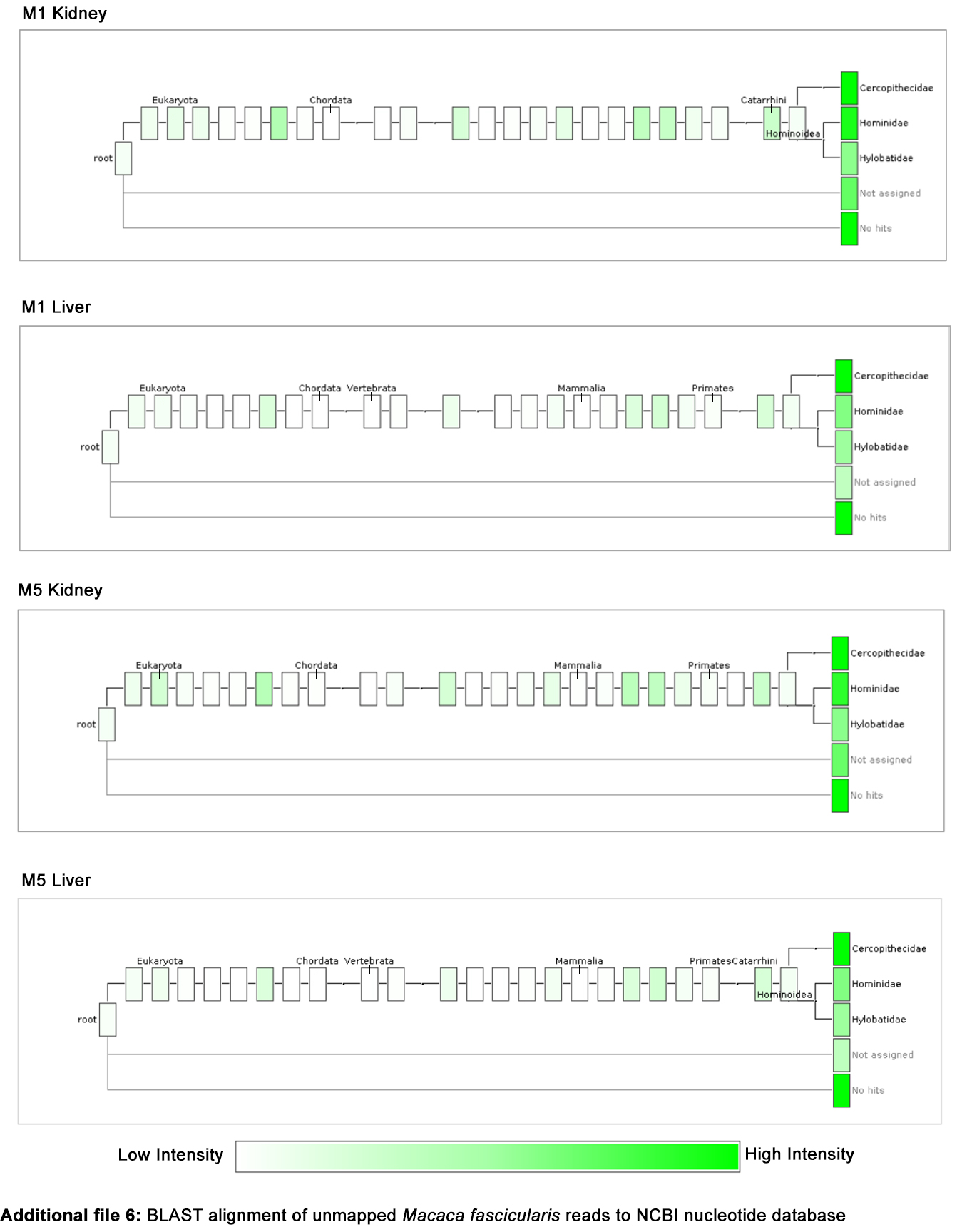

Supplement: Supplementary file 6 — Additional file 6. BLAST alignment of unmapped Macaca fascicularis reads to NCBI nucleotide database. Graphical representation of unmapped M. fascicularis sequence reads assigned to taxonomic ranks based on BLAST alignment with NCBI nucleotide (nt) database. Intensity of the colour green represents the number of unmapped reads mapped to a respective taxonomic rank—the more intense the green, the higher the number of reads assigned to a particular taxon. [file 13104_2018_4014_MOESM6_ESM.jpg]
